# Retraction: Impact of Dual Antiplatelet Therapy Versus Monotherapy in Acute Stroke Management: A Systematic Review

**DOI:** 10.7759/cureus.r176

**Published:** 2025-04-01

**Authors:** Muhammad Adil Areeb, Rakesh Mohanty, Hiren B Hisoriya, Javeria Mithani, Oluwatobiloba T Ogungbemi, Kesha Pathak, Umar Farooq, Rauda Al Dhaheri, Ahmad Zubair Aziz, Nishath Kausar, Ali Raza

**Affiliations:** 1 Neurology, Avicenna Tajik State Medical University, Dushanbe, TJK; 2 Anesthesiology, King's College Hospital, London, GBR; 3 Internal Medicine, Poona Hospital and Research Centre, Pune, IND; 4 Internal Medicine, Karachi Medical and Dental College, Karachi, PAK; 5 Internal Medicine, Caucasus's International University, Tbilisi, GEO; 6 Internal Medicine, Gujarat Adani Institute of Medical Sciences, Bhuj, IND; 7 Medicine and Surgery, Avicenna Medical College, Lahore, PAK; 8 Cardiology, Dubai Medical College for Girls, Dubai, ARE; 9 Internal Medicine, Hunan Normal University, Khanpur, PAK; 10 Internal Medicine, Sitara Medical Center, Hyderabad, IND; 11 Internal Medicine, Nishtar Medical University, Multan, PAK

The Editors-in-Chief have retracted this article. An investigation by the journal found evidence of authorship manipulation. The Editors-in-Chief therefore no longer have confidence in the provenance and reliability of the article contents.

